# Immune Mediated Degeneration and Possible Protection in Glaucoma

**DOI:** 10.3389/fnins.2019.00931

**Published:** 2019-09-02

**Authors:** Teresa Tsai, Sabrina Reinehr, Ana M. Maliha, Stephanie C. Joachim

**Affiliations:** Experimental Eye Research, University Eye Hospital, Ruhr-University Bochum, Bochum, Germany

**Keywords:** glaucoma, complement system, autoantibody, organ culture, porcine, neuroprotection

## Abstract

The underlying pathomechanisms for glaucoma, one of the most common causes of blindness worldwide, are still not identified. In addition to increased intraocular pressure (IOP), oxidative stress, excitotoxicity, and immunological processes seem to play a role. Several pharmacological or molecular/genetic methods are currently investigated as treatment options for this disease. Altered autoantibody levels were detected in serum, aqueous humor, and tissue sections of glaucoma patients. To further analyze the role of the immune system, an IOP-independent, experimental autoimmune glaucoma (EAG) animal model was developed. In this model, immunization with ocular antigens leads to antibody depositions, misdirected T-cells, retinal ganglion cell death and degeneration of the optic nerve, similar to glaucomatous degeneration in patients. Moreover, an activation of the complement system and microglia alterations were identified in the EAG as well as in ocular hypertension models. The inhibition of these factors can alleviate degeneration in glaucoma models with and without high IOP. Currently, several neuroprotective approaches are tested in distinct models. It is necessary to have systems that cover underlying pathomechanisms, but also allow for the screening of new drugs. *In vitro* models are commonly used, including single cell lines, mixed-cultures, and even organoids. In *ex vivo* organ cultures, pathomechanisms as well as therapeutics can be investigated in the whole retina. Furthermore, animal models reveal insights in the *in vivo* situation. With all these models, several possible new drugs and therapy strategies were tested in the last years. For example, hypothermia treatment, neurotrophic factors or the blockage of excitotoxity. However, further studies are required to reveal the pressure independent pathomechanisms behind glaucoma. There is still an open issue whether immune mechanisms directly or indirectly trigger cell death pathways. Hence, it might be an imbalance between protective and destructive immune mechanisms. Moreover, identified therapy options have to be evaluated in more detail, since deeper insights could lead to better treatment options for glaucoma patients.

## Introduction

Glaucoma is a multifactorial and neurodegenerative disease, which is characterized by a chronic loss of retinal ganglion cells (RGCs) and their axons (Casson et al., 2012; EGS, 2017). Patients suffer from irreversible visual field loss, which ultimately leads to blindness (Stevens et al., 2013). As a result, glaucoma is one of the most common causes of blindness worldwide, affecting approximately 79.6 million people by 2020 (Quigley and Broman, 2006; EGS, 2017). As society ages, there will be an additional increase in severe visual impairment and blindness and by the year 2030 nearly 13% of these patients will be affected by glaucoma (Finger et al., 2011; Lang, 2014).

Glaucoma can be differentiated into several types, which makes diagnosis difficult. Hence, in most western countries around 50% of patients with manifest glaucoma are unaware of their disease (Tielsch et al., 1991; Mitchell et al., 1996; Grodum et al., 2002; Quigley and Jampel, 2003; EGS, 2017). Additionally, in most cases, glaucoma is diagnosed too late (Martus et al., 2005), since it might be clinically not detectable until 20–40% of RGCs are lost, resulting in a potential 10 year delay in diagnosis (Zeyen, 1999; Kerrigan-Baumrind et al., 2000). In general, glaucoma can be subdivided into primary and secondary forms. The latter usually occurs as a result of an already existing (eye) disease or as an undesirable side effect of drugs, after a medical procedure, as well as after traumatic injury. The most common form of primary glaucoma is the primary open-angle glaucoma (POAG). POAG is generally characterized by a clinical triad of elevated intraocular pressure (IOP), the appearance of optic atrophy, and a progressive loss of peripheral visual sensitivity in the early stages of the disease, which may ultimately progress and then impair visual acuity (Quigley, 1993). But in about 30% of all cases, glaucomatous damage is developed IOP-independently (Sommer et al., 1991). This form is known as normal-tension glaucoma (NTG). It is a controversial issue if the separation of POAG and NTG is artificial and both diseases trace back to the same pathogenic mechanisms.

The underlying pathomechanisms for glaucoma are still not fully identified. High IOP remains the main risk factor. In addition, age, myopia, gender, and ethnicity seem to play an important role in the development of glaucoma (Coleman and Miglior, 2008; Chen et al., 2012; McMonnies, 2017). For example, people of African descent are more likely to develop POAG than Caucasians, and Asians are particularly prone to NTG (McMonnies, 2017). Although the exact pathomechanisms of glaucoma are still unclear, several possible factors that likely contribute to the onset of glaucoma are discussed. In addition to mechanical processes, circulatory disorders, excitotoxicity, and immunological reactions are also considered contribute to the pathogenesis of glaucoma (Joachim et al., 2005; Casson, 2006; Tezel et al., 2010; Evangelho et al., 2019). Moreover, hypoxic processes as well as oxidative stress are involved in the early disease progression (Zanon-Moreno et al., 2008; Greco et al., 2016).

Currently, an elevated IOP is considered the main risk factor and can be treated medically or surgically. Therefore, IOP lowering is the main treatment option and known to slow down or even stop progressive vision loss in patients (Vass et al., 2007). Although the IOP in NTG is not significantly increased, lowering pressure is the common therapy. For every glaucoma patient, an individual desired IOP is determined, depending on individual disease factors. Unfortunately, in many cases, despite medical or surgical IOP lowering therapy, optic nerve and RGC degeneration as well as visual field loss continue on a long-term basis (Chang and Goldberg, 2012; Pascale et al., 2012). Thus, it would be tremendously beneficial to develop treatment options that protect RGCs and preserve visual function through mechanisms other than IOP reduction. In the last years, researchers have searched for pharmacological or molecular genetic methods to protect retinal neurons or nerve fibers and thus prevent cell death. The list of neuroprotective substances studied so far is long. Unfortunately, a big breakthrough has not yet been achieved. Recent research approaches, however, give hope and point to new and promising therapeutic strategies.

## Immune Response in Glaucoma Patients

In recent years, possible pathogenic factors, such as oxidative stress (Tezel et al., 2010; Tezel, 2011), ischemic events (Almasieh et al., 2012; Schmid et al., 2014), or increased glutamate levels (Dreyer et al., 1996; Neufeld et al., 1997; Kuehn et al., 2017b), were implicated to contribute to glaucoma. Furthermore, a possible involvement of the immune system moved more and more into the focus (Tezel and Wax, 2004; Grus et al., 2008; Wax, 2011). In patients with POAG as well as with NTG, changes in the antibody profile were found in serum and aqueous humor (Grus et al., 2004; Joachim et al., 2007; Boehm et al., 2012). One of the first antibodies identified was against heat shock protein (HSP) 60 and small HSPs (Wax et al., 1994). Further studies revealed complex altered antibody responses in patients. Some of these antibodies were upregulated, such as HSP27 (Tezel et al., 1998), HSP70 (Joachim et al., 2007), γ-enolase (Maruyama et al., 2000), α-fodrin (Grus et al., 2006), or myelin basic protein (Joachim et al., 2008). Further analysis of the increased antibody titers showed that the direct administration of small HSPs to retinal tissue or cells can induce cell death through apoptotic mechanisms. Thus, increased titers of circulating antibodies HSPs, like HSP27, may appear pathogenic in some patients (Tezel et al., 1998). However, a downregulation of antibodies, like GFAP, vimentin, β-crystallin, or 14-3-3 was also detected (Joachim et al., 2007, 2008; Bell et al., 2015). Since some autoantibodies have neuroprotective potential on neuronal cells, the reduction of GFAP and 14-3-3 appears to be an indication of the loss of naturally occurring protective autoimmunity (Bell et al., 2013). Similar antibody changes were noted in other neurodegenerative diseases, such as Alzheimer’s disease and multiple sclerosis (Krumbholz et al., 2012; Liao et al., 2013). Patients with Alzheimer’s disease for example exhibited a reduced level of their protective autoantibodies against amyloid-β (Dodel et al., 2011) whereas patients with multiple sclerosis showed an upregulation of demyelinating autoantibodies (Elliott et al., 2012).

Furthermore, depositions of IgG antibodies were found in the retinae of glaucomatous eyes (Wax et al., 1998; Gramlich et al., 2013). Antibodies are usually able to activate the complement system, a part of the innate immune response. This could also be the case in glaucoma. Here, elevated complement proteins, such as C3 or lectin pathway associated proteins, were noted in the sera and retinae of POAG patients (Boehm et al., 2010; Tezel et al., 2010). Also, evidences showed altered macroglia reactions as well as contributions of activated microglia cells (Yuan and Neufeld, 2001; Wang et al., 2002). Recently, Chen et al. reported that an IOP elevation can induce infiltration of autoreactive T-cells into the retina, which cause neurodegeneration by cross-reacting with HSP-expressing RGCs. Furthermore, they noted that both POAG and NTG patients also have an increase of HSP27- and HSP60-specific T-cells, indicating that these findings are likely to be of relevance for glaucoma patients (Chen et al., 2018).

## Findings From Glaucoma Animal Models

### Immune Response in Glaucoma Models

In order to analyze the altered immune response found in human glaucoma patients more precisely, an experimental autoimmune glaucoma (EAG) model was developed (Wax et al., 2008). This model should help to shed light on the question if the immune system alterations are cause or consequence of the disease. Glaucomatous-like damage in this model is induced by immunization with ocular antigens without altering IOP. The immunization with antigens, like HSP27, HSP60, or S100B protein lead to RGC loss and optic nerve degeneration after 28 days (Wax et al., 2008; Casola et al., 2015; Noristani et al., 2016; Reinehr et al., 2018a). Also, immunization with an optic nerve antigen homogenate (ONA) provoked glaucoma-like damage in the animals (Laspas et al., 2011; Noristani et al., 2016). However, prior to the loss of RGCs in this model, it was possible to detect antibody deposits in the retina, similar to those seen in tissue from glaucoma patients (Joachim et al., 2012). IgM deposits were already detected after 7 days, while IgG deposits could be detected in the ganglion cell layer of immunized animals after 14 days. These deposits were often co-localized with apoptotic RGCs (Joachim et al., 2014). In an intermittent ocular hypertension (OHT) animal model, IgG autoantibody deposits and microglia activation were also notable. Furthermore, elevated serum autoantibody immunoreactivities were detected, for example against glutathione-S-transferase and transferrin (Gramlich et al., 2016).

The findings of the antibodies raised the question, how they are contributing to glaucomatous cell death. It is possible that antibodies activate certain pathways, like the complement system. It is known that IgGs are able to initiate the complement cascade (Sontheimer et al., 2005; Ehrnthaller et al., 2011). In the EAG model, an activation of the complement system was noted via the lectin pathway in the retina and the optic nerve at the early stage of the disease, already 2 weeks after the immunization. The number of RGCs was still unchanged at this time (Reinehr et al., 2016a). Interestingly, in addition to the upregulation of complement factors, an increased number and activation of microglial cells could be observed in the EAG model at this early stage (Noristani et al., 2016).

### Activation of the Complement System

**FIGURE 1:**
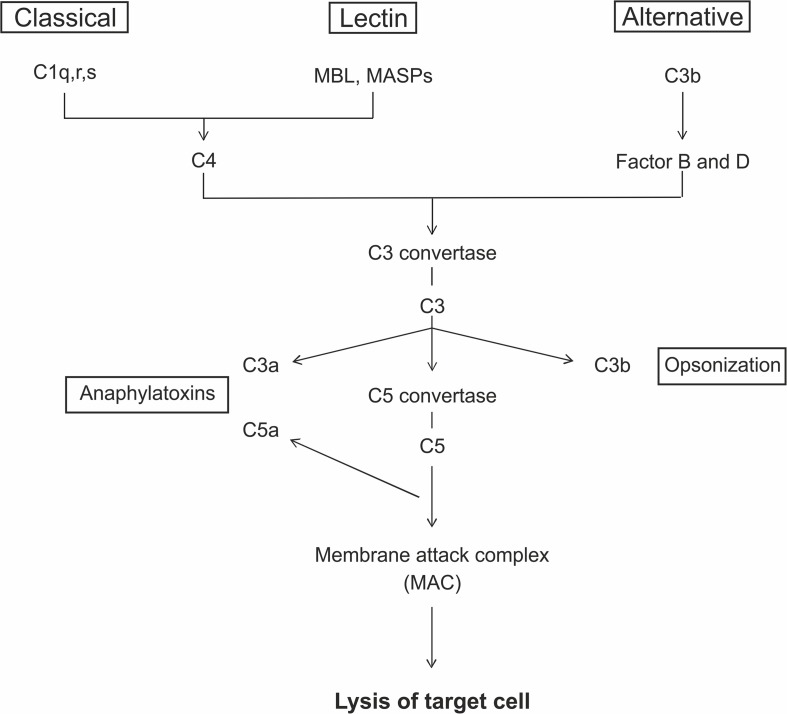
The complement system can be activated via three different pathways. The classical pathway is initiated by antibody complexes binding to C1q. The mannose binding lectin (MBL) and the mannose-associated-serine-proteases (MASPs) bind to specific carbohydrate structures leading to the activation via the lectin pathway. The alternative pathway is spontaneously activated through the cleavage of C3 to C3b. All three pathways lead to the generation of C3 convertases that cleaves the C3 protein into C3a and C3b. C3b acts in the opsonization of target cells and additionally form the C5 convertase, which cleaves C5 to C5a and C5b. C5a and C3a act as anaphylatoxins. At the end, the interaction of C5b with C6, C7, C8, and C9 lead to the formation of C5b-9, the membrane attack complex (MAC). MAC is the terminal pathway, which can cause lysis of the target cells due to a formation of a pore.

The complement system is part of the innate immune response and consists of a large number of different plasma proteins that interact to opsonize pathogens and to induce a series of inflammatory responses. It is also a bridge between the innate and the adaptive immunity. The proteins are mostly synthesized in the liver and exist in the plasma or on cell surfaces as inactive precursors, called zymogens (Nesargikar et al., 2012). The activation is initiated through a triggered enzyme cascade. A key site for the activation processes is the pathogen surface and there are three distinct pathways leading to complement activation, the classical, the lectin, and the alternative pathway (Figure 1). The classical pathway plays a role in both innate and adaptive immune response. The first component, C1q, can bind either to antibodies complexed with antigens or to naturally produced antibodies. The lectin pathway can be induced through the mannose binding lectin (MBL). It binds specifically to sugar residues, which are present on many pathogen surfaces. MBL forms a complex with the mannose-associated-serine-proteases 1 and 2 (MASP1- and 2), which then activate the further complement cascade. The third pathway, the alternative one, is activated spontaneously via hydrolysis of C3 into C3b (Murphy and Walport, 2008). Finally, the membrane attack complex (MAC) is formed. MAC has a hydrophobic external face and a hydrophilic internal channel. The disruption of the lipid bilayer leads to the loss of cellular homeostasis, which results in the lysis of the target cell (Fosbrink et al., 2005; Murphy and Walport, 2008).

As already described in glaucoma patients, also in animal models a dysregulation of the complement system seems to be involved in disease development. Significantly more complement depositions were described in various OHT studies. For example, C3 and MAC depositions were found in rat retinae 14 and 28 days after OHT induction (Kuehn et al., 2006). Increased C3 and MAC levels were also observed 6 weeks after IOP elevation through laser treatment (Jha et al., 2011). In addition, even a moderate increased IOP, of about 19%, leads to an enhancement of the complement factors C3 and MAC in the retinae of rats (Becker et al., 2015). To investigate the contribution of the complement system in glaucoma independent from IOP, studies on the EAG model were carried out. It is known that a loss of RGCs is observable 28 days, but not 14 days after immunization with ONA (Laspas et al., 2011; Noristani et al., 2016; Reinehr et al., 2016b). Hence, the question arises whether an activation of the complement cascade is detectable even before cell death. Significantly more C3 depositions were noted in retinae and optic nerves of ONA immunized animals after 7 days. Furthermore, the terminal pathway of the complement system, MAC, was also activated at these points in time. Due to previous findings of IgG antibodies in human (Gramlich et al., 2013) and animal glaucomatous eyes (Joachim et al., 2014), activation of the complement cascade via C1q seemed likely. Interestingly, this was not the case. In human glaucoma as well as in OHT models, on the other hand, elevated C1q levels were identified (Kuehn et al., 2006; Stasi et al., 2006; Howell et al., 2011). Also, an inhibition of C1q seems to be protective against dendritic and synaptic degeneration (Williams et al., 2016). In previous studies using the EAG model, depositions of IgG were noted at 14 days, but not at 8 days after immunization (Joachim et al., 2014). Hence, it is possible that C1q is expressed at later points in time in this model. Interestingly, a simultaneous activation of components of the lectin pathway in retinae and optic nerves was observed. The lectin pathway cannot only be initiated through mannose residues on pathogen surfaces, but also through apoptotic and necrotic cells (Ogden et al., 2001; Nauta et al., 2003; Stuart et al., 2005). It is also known that hypoxia induces restructuring of the endothelial cell surface, resulting in an activation of the complement system via the lectin pathway (Collard et al., 1999). As stated above, in glaucoma human donor eyes, proteomic analysis revealed an upregulation of proteins linked to the lectin pathway, such as MASP1 and MASP2 (Tezel et al., 2010). It is known that MASP2 cleaves C4 and C2 to form the C3 convertase. MASP1 alone is insufficient to activate the lectin pathway, but the activation of both MASPs ultimately initiates the complement cascade (Matsushita et al., 2000; Takahashi et al., 2008). After immunization with the glia protein S100B, an activation of the lectin pathway occurred. Here, MBL was upregulated in the S100B retinae after 3 days, and after 7 and 14 days in optic nerves as well (Reinehr et al., 2018b).

### Role of Microglia in Glaucoma Models

The observations of complement proteins in glaucomatous retinae and optic nerves raise the question how these components could enter the eye. Although the blood-retina-barrier is not impenetrable, most proteins cannot invade the eye. Therefore, local synthesis by resident cells in the retina is necessary. It seems likely that glia cells are the source of complement components. Microglia are the resident immune cells in the central nervous system and therefore also in the retina (Kettenmann et al., 2011; Karlstetter et al., 2015). In the retina, they are mainly located in the ganglion cell layer or in the inner plexiform layer. In the optic nerve, activated microglia are first localized in the optic nerve head (Bosco et al., 2011). Microglia are linked to many neurodegenerative diseases, such as multiple sclerosis (Ajami et al., 2011), Alzheimer’s disease (Fuhrmann et al., 2010), and Parkinson’s disease (Ouchi et al., 2005). Activated microglia are also a hallmark in retinal diseases, including diabetic retinopathy (Zeng et al., 2008) or uveitis (Rao et al., 2003; Kerr et al., 2008). When neurons are damaged, microglia respond by adopting an activated phenotype (Kreutzberg, 1995; Graeber and Streit, 2010; Ramirez et al., 2017). Additionally, they can change the expression of different enzymes, receptors, cytokines, and growth factors (Rojas et al., 2014). In the EAG model, a significantly higher number of microglia was seen in the retina after 14 and 28 days when immunizating with different ocular antigens (Casola et al., 2016; Noristani et al., 2016). But not only the number of these cells was increased. In addition, more activated cells were observed in these animals, especially 14 days after immunization. However, no alterations could be detected anymore at 28 days (Noristani et al., 2016). These results are in accordance with OHT studies, where a microglia activation was noted prior to RGC loss (Ebneter et al., 2010; Bosco et al., 2011). In a laser induced OHT model, a non-proliferative microglia activation was detected already after 24 h (de Hoz et al., 2018). Also, after an intravitreal application of S100B, an increase in the microglia cell number was accompanied with a loss of RGCs after 14 days (Kuehn et al., 2018).

It is known that the transcription factor nucleus factor-kappa-light-chain enhancer of activated B cells (NFκB) controls the migration of microglia to the site of injury due to expression of β-integrin CD11a. In rats, which were systemically immunized with S100B, an increase of NFκB could be observed in retinae after 7 and 14 days. Furthermore, enhanced levels of the pro-inflammatory cytokine IL-1β were observed in aqueous humor of S100B animals at day 7 (Reinehr et al., 2018b). Yoneda et al. noted that IL-1β plays an important role in mediating ischemic and excitotoxic damage in glaucomatous retina (Yoneda et al., 2001). Several studies claim that IL-1β is secreted by microglia after photo-oxidative damage (Hu et al., 2015; Jiao et al., 2015; Natoli et al., 2017), in neovascular age-related macular degeneration (Lavalette et al., 2011), in retinitis pigmentosa (Zhao et al., 2015), and after retinal detachment (Kataoka et al., 2015). Besides microglia/macrophages, also NFκB was reported to induce transcription of the IL-1β gene (Cogswell et al., 1994).

### The Immune System as Therapeutic Target in Glaucoma

Several findings demonstrate a contribution of the immune system in glaucoma pathogenesis. Since lowering the IOP is the common treatment approach for glaucoma to date, new therapeutic solutions are needed. As noted, previous studies in glaucoma models discuss the role of the complement system for glaucoma pathology. Therefore, the inhibition of it could be a potential therapeutic target. In an OHT model, it could demonstrate that the cobra venom factor (CVF) depleted the complement system and led to a reduced loss of RGCs due to inhibition of intrinsic and extrinsic apoptotic pathways. Furthermore, the treatment with CVF resulted in a diminution of MAC depositions (Jha et al., 2011). Additionally, C5 deficient glaucomatous DBA/2J mice exhibited reduced neurodegeneration in comparison to C5-sufficient animals. Inhibition of complement activation was accompanied by reduced MAC deposition and RGC loss (Howell et al., 2013). Recently, Bosco et al. published a retinal gene therapy approach, where they injected the C3 inhibitor CR2-Crry intravitreally in DBA2/J mice. They revealed a reduction of C3d in RGCs and the inner retinal layers leading to a preservation of RGC somata and axons (Bosco et al., 2018). These results demonstrate the possibility of a complement inhibition for glaucoma treatment.

In terms of microglial inhibition, several studies investigated how minocycline effects microglia in glaucoma models. This semi-synthetic tetracycline can cross the brain-blood-barrier, respectively, the retina-blood-barrier. In neurodegenerative conditions accompanied with neuroinflammation, such as multiple sclerosis or Parkinson’s disease, remarkable neuroprotective effects were noted (Kim and Suh, 2009; Russo et al., 2016). In a study, where rats received an intravitreal injection of S100B and were additionally treated with minocycline, loss of RGCs was diminished and a preservation of the optic nerve structure was demonstrated (Kuehn et al., 2018). In OHT models it has been observed that after treatment with either minocycline or a high dose of irradiation, microglia activation was significantly reduced and hence less RGC death was noted (Levkovitch-Verbin et al., 2006; Bosco et al., 2008; Bosco et al., 2012). Minocycline not only prevented the increase of Iba1^+^ microglia, but also decreased the GFAP^+^ area and preserved the anterograde transport after OHT (Bordone et al., 2017).

All these promising results underline a contribution of the immune system in glaucoma disease. Nevertheless, more studies are needed to bring these aspects from bench to bedside.

## Different Models for Screening of Neuroprotective Agents

**FIGURE 2:**
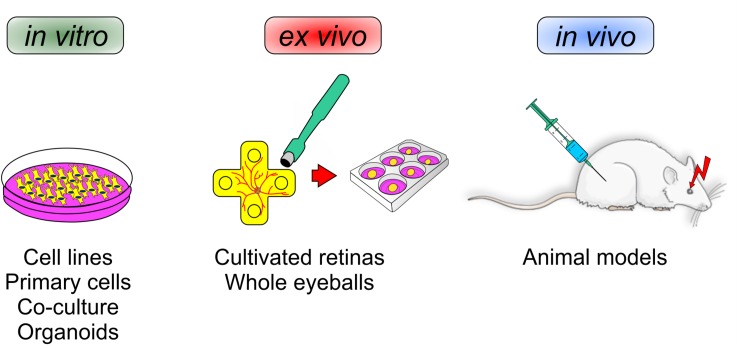
Various mechanisms can influence a loss of retinal ganglion cells. To identify novel neuroprotective treatments for glaucoma, different experimental setups are currently used. *In vitro* analyses reveal the function of new therapeutics on single cells, mixed cultures, or organoids. *Ex vivo* experiments can provide insights into the whole retina, e.g., in cultured porcine/bovine retina. *In vivo* investigations in animals have the advantage to provide a closer look at local and systemic mechanisms and possible side effects.

In the following section, we will discuss and elaborate different existing models that are suitable to investigate neuroprotective agents. To this end, this section deals with the pros and contras of different *in vitro* cell lines, primary cells, co-culture systems, as well as organoids. Also, different *in vivo* animal models will be discussed. In addition, an alternative model, namely explanted and cultured retinas of different animals, like pigs and cows, will be introduced (Figure 2).

### Findings From *in vitro* Cell Culture Studies

Since the underlying molecular pathomechanisms occurring in glaucoma are not fully understood yet, standard therapeutic interventions deal with the deceleration of disease progression and target the main risk factor, namely the elevated IOP. The most common medical therapy for glaucoma are IOP lowering eye drops, which include prostaglandin analogs, beta-blockers, diuretics, cholinergic agonists, and alpha agonists (Narayanaswamy et al., 2007; Conlon et al., 2017). The mechanism of action of those classes of eye drops are different. The most commonly used classes are prostaglandin analogs. An increase of the aqueous humor outflow results in a decreased IOP (Gaton et al., 2001). Since the medical therapy does not always reduce the IOP sufficiently, other therapy options such as laser treatments and surgical interventions are performed to lower IOP. Laser treatments aim to reduce the IOP in a less invasive manner than e.g., trabeculectomy or drainage implants (Latina et al., 1998; Conlon et al., 2017).

For the evaluation of novel therapies, it is inevitable to have models that on the one hand cover underlying pathomechanisms and on the other hand allow the screening of new therapeutic approaches. Monoclonal *in vivo* cultured cells or cell-lines are, in general, commonly used models for research of several pathomechanisms involved in eye diseases. There are many cell-lines obtained from retinal tissue, like retinal pigment epithelium cells (Liu et al., 2016), retinal microvascular endothelial cells (Xie et al., 2017) and retinal cone photoreceptor cells (Sanchez-Bretano et al., 2017). For example, with the help of the human retinal pigment epithelial cell line ARPE-19, it has recently been shown that baicalin, a flavonoid extract from *Scutellaria baicalensis*, protects against high glucose-induced cell injury such as it occurs in diabetic retinopathy (Dai et al., 2019). Also, cobalt-chloride (CoCl_2_) damaged ARPE-19-cells were protected by betulinic acid, a pentacyclic triterpenoid with anti-oxidative effects (Cheng et al., 2019). On the other hand, several studies investigate the protective, therapeutic effect of RNA-modulation on degenerative RGCs (Nickells et al., 2017; Yu et al., 2019).

Due to the structure of the retina, which consists of different cell-enriched layers and layers with synaptic connections, homeostasis and interactions of retinal cells are crucial for its integrity and visual signal transduction (Hoon et al., 2014; Grossniklaus et al., 2015). Cell-lines as well as primary monoclonal cultured cells, consisting of only one retinal cell type, are not able to mimic the *in vivo* situation of the retina at all. Furthermore, cell-lines are immortalized which on the one hand simplifies the handling but on the other hand requires manipulated/modified DNA. Modifications of DNA can often be accompanied by further unintended gene alterations. A very prominent example for a cell-line with undefined DNA modifications are RGC-5 cells. RGC-5 cells have been used for researches on RGCs and were introduced as a cell-line derived from rat RGCs (Krishnamoorthy et al., 2001). The expression of RGC-characteristic proteins like Brn-3a or Thy1 was given, but over time many concerns of several laboratories raised, since it was noted that the cells seem to be of murine origin and expressions of several not-RGC-characteristic proteins were observed (Wood et al., 2010; Sippl and Tamm, 2014). The ambiguity of the RGC-5 cell-line as well as the fact that the visual system benefits from the interaction of several retinal cell types, indicates that this cell-line is possibly not the best model for glaucoma research.

Besides, there are several available *in vitro* models of primary mixed cultures of retinal tissue. Li et al. (2015) established a co-culture system of Sprague-Dawley rat retinas together with microglia and Müller cells to evaluate the effect of interactions between microglia and Müller cells on the photoreceptor cell survival. Another model used for investigations on retina are retinal organoids. In those models the goal of research is more the improvement of co-culture systems to investigate retina-RPE dynamics during retinal development. A study by Akhtar et al. (2019) noted that the co-culture of different staged murine RPE cells accelerated photoreceptor differentiation of retinal organoids derived from human-induced pluripotent stem cells. Newly developed mouse multipotent retinal stem cell-derived RGCs, which expresses characteristic RGC-genes, are a suitable model to investigate RGC-aimed gene delivery systems for neuroprotective agents, such as non-viral neurotrophic factor gene therapy (Chen et al., 2019).

### Treatment Screening in *in vivo* Models

Most frequently used models for research in general, as well as for ophthalmic research, including glaucoma, are animal models. The first form of retinal degeneration inherited in a mouse model was reported around 90 years ago (Keeler, 1924). Since then, the usage of mouse models for retinal degeneration by genetic modifications increased (Dalke and Graw, 2005; Baehr and Frederick, 2009). Genetics in vertebrates are highly correlated. Especially retinal structure and function of rodents are very similar to those of human: the neuronal cells of the retina and the cell body as well as the synapse distribution and connectivity is comparable in all vertebrate retinae (Hoon et al., 2014). Due to those facts and the short life cycle of rodents, especially mice, make them suitable and very common models for ophthalmic research. Furthermore, the modifications of several genes, to obtain knock-in or knock-out-based diseases, is easy to manage and enables a wide area for diverse research. Inbreeding of animals prevents genetic variability within the mouse strain, which guarantees an equal genetic background of the animals during experiments. Based on this, glaucomatous models, such as the DBA/2J mouse, are used to test new therapeutic approaches such as the flavonoid fisetin. This treatment results in retention of retinal function by suppressing inflammatory response (Li et al., 2019).

Besides the high effort for the bureaucracy, breeding and housing of animals involve higher costs than cell cultures. Despite the genetic similarities within the retina of vertebrates, there are broad disparities between the structure of human and rodent eyes (Zhou et al., 2007). Not only the size of the eyes differs strongly, also the anatomy of the retina varies. Due to the highest density of cone photoreceptors the fovea centralis, which is located in the center of the macula, is responsible for sharp central vision in humans (Curcio et al., 1990). Rodents, on the other hand, do not have a macula, which makes the research of e.g., age-related macular degeneration much more complicated (Volland et al., 2015). Also, the distribution of rods and cones in the mice retina differs from the human retina. A further difference between the anatomy of rodent and human eyeballs in general is that rodents do not have a real vitreous body: the primary vitreous body recedes completely on postnatal day 30, whereas the secondary vitreous body develops on postnatal day four (Tkatchenko et al., 2010). The lens of rodents is, in comparison to that of humans, much bigger since it fills the whole eye to stabilize it.

In the last few years, many theories for different pathomechanisms leading to glaucoma were discussed. Several rat glaucoma models indicate that the shortage of neurotrophic factors, like BDNF or NGF, in the optic nerve might contribute to the progression of glaucomatous optic nerve degradation (Song et al., 2015). Studies in regard to neurotrophic factors indicate that this might be of interest for glaucoma treatment. The injection as well as the pre-treatment with BDNF lowered RGC loss and suppressed axon loss of glaucomatous rats (Ko et al., 2001; Martin et al., 2003). Another pathomechanism, which seems to be involved in glaucoma disease, is excitotoxicity (Song et al., 2015). To this end, the blockage of excitotoxicity might also be of interest for glaucoma treatment. A well-researched NMDA-receptor antagonist is MK801, which was shown to lower RGC death rate in different glaucoma rat models (Chaudhary et al., 1998; Nucci et al., 2005).

Looking for alternative models, where no classical animal experiments are needed, but similarities to human tissue are still given, it becomes clear that porcine or bovine tissue might serve as a good option.

### *Ex vivo* Organ Culture Models

Due to the high similarities between bovine or porcine and human vision as well as their retinal structure, these retinae seem to be a very promising alternative to animal experiments in ophthalmologic research. An advantage of these eyes is that they are more similar to human eyes than those of rodents. Not only the size is comparable between porcine/bovine and human eyeballs, also the vision, especially of pigs, is more likely to the vision of humans. Humans are trichromats and their cones contain of three different subtypes due to their activation through different wavelengths: they are divided into the short (S)-, the middle (M)-, and the long (L)-cones, depending on the wavelength-sensitivity of the opsins (Nathans et al., 1986). Mice, in contrast, contain of a dichromatic vision, expressing M-cones and ultraviolet-cones (Jacobs et al., 1991). As mice, pigs also have a dichromatic vision, but still the porcine retina and therefore the vision is more like the human vision: porcine cone photoreceptors contain of two opsins, the S- and the M-cones, (Szel et al., 1988; Li et al., 1998; Hendrickson and Hicks, 2002). Even though pigs do not have a real macula, they still have a part in the retina which is very similar to the human macula. This area is called visual streak and is located above the optic disc extending from nasal to the almost temporal edge (Hendrickson and Hicks, 2002). In contrast to classical animal testing, the bureaucracy to use porcine or bovine eyes is much less. In addition, the costs are lower, because porcine and bovine eyes can be obtained from local slaughterhouse, where they are a waste product of the food industry.

Another important advantage of the usage of porcine or bovine retina is, that in contrast to conventional cell culture models, the retina itself can be cultivated for a certain time. The retinal organ culture allows the maintenance of interactions and connections of neurons within the retina. Of course, cultivating retinal organ explants has a time limitation, since the retina, due to the axotomy and the removal of retinal pigment epithelium, cannot be kept alive for a long period *ex vivo*. Nevertheless, during cultivation the nutrient supply can be maintained chemically to alleviate degeneration processes.

Glaucoma is a multifactorial disease where the exact pathomechanisms are not fully understood yet, but it is known that also hypoxic processes as well as oxidative stress are involved in the early progression (Zanon-Moreno et al., 2008; Greco et al., 2016). Chemical substances, such as hydrogen peroxide (H_2_O_2_) and CoCl_2_ can be used *in vitro* to simulate this oxidative stress or hypoxic processes (Hurst et al., 2017; Kuehn et al., 2017a). The combination of porcine retina organ culture and chemical simulation of degenerative pathomechanisms *in vitro* is a very well-suited alternative model for ophthalmic research. A commonly used substance, as mentioned above, to mimic oxidative stress, not only in retinal tissue, is CoCl_2_. It is used for the investigation of the mentioned pathomechanisms as well as possible treatments against it by using several cells of different origin like mesenchymal cells (Yoo et al., 2016), PC12 cells (Hartwig et al., 2014), RPE cells (Li et al., 2013; Cheng et al., 2019), as well as in retinal organ culture (Kuehn et al., 2017a; Maliha et al., 2019). A substance which is frequently used to induce oxidative stress is H_2_O_2_. This allows the investigation of underlying pathomechanisms and possible neuroprotective substances for various disorders, such as retinal diseases (Cui et al., 2017; Du et al., 2018; Zhao et al., 2019). As shown in previous studies of our group, the addition of H_2_O_2_ as well as CoCl_2_ leads to strong neurodegenerative effects in the inner retinal layers of porcine retinae (Hurst et al., 2017; Kuehn et al., 2017a).

**FIGURE 3:**
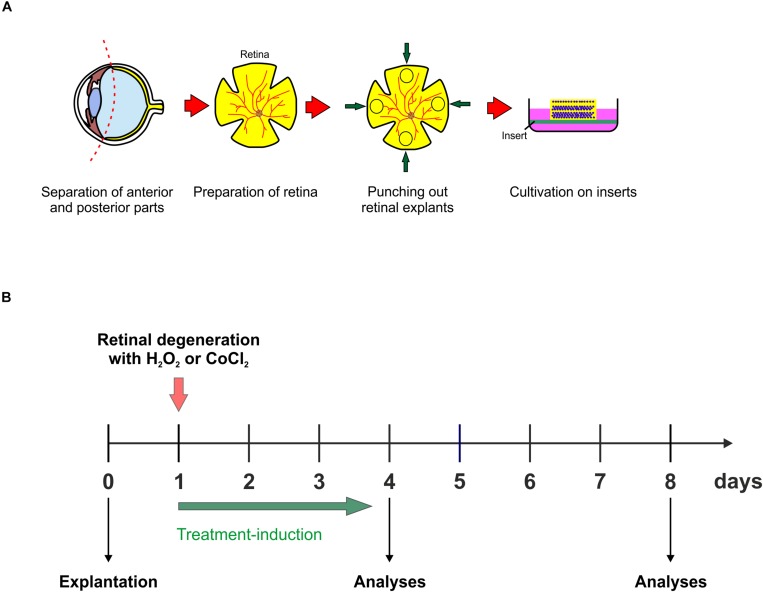
**(A)** Schematic illustration of the explantation process. Porcine eyes were obtained from the local slaughterhouse. Anterior parts of the eyeball were separated from the posterior, retina containing part of the eye. A dermal punch was used to punch out four retinal explants per eye and cultivated on an insert the ganglion cell layer facing up. **(B)** Retinal explants were cultivated in 6-well plates for up to eight days at 37°C and 5% CO_2_. There are two possibilities to induce different pathomechanisms in the retina, which lead to a degeneration of the inner layers. Either with 300 μM H_2_O_2_ for 3 h to induce oxidative stress on day one of cultivation or with 300 μM CoCl_2_ for 48 h, starting on day one, to induce hypoxic processes. The best point in time to start therapeutic treatment is at day one, simultaneously to the degeneration via H_2_O_2_ or CoCl_2_. The treatment-duration varies, depending on the substance. In the end of the cultivation, retinal explants can be prepared for e.g., immunohistological and qPCR-analyses.

For the preparation of a porcine retina organ culture, as mentioned above, porcine eyes are obtained from the local slaughterhouse. Surrounding tissue as well as anterior parts of the eye, are separated from the posterior part of the eye, including the retina. Using a dermal punch, retinal explants are punched out and placed, with the ganglion cell layer facing up, on a millicell insert. The retina is than placed in a 6-well-plate and can be cultivated for up to eight days (Figure 3A; Hurst et al., 2017; Kuehn et al., 2017a; Maliha et al., 2019). Simulation of oxidative stress in porcine retina organ culture can be achieved through the addition of H_2_O_2_ for 3 h at the first day of cultivation (Figure 3B). Hypoxic processes, however, can be simulated in porcine retinae, by adding CoCl_2_ to the medium for 48 h, from day one to day three of cultivation (Figure 3B). CoCl_2_ as well as H_2_O_2_ induce strong degenerative effects in retinal tissue, however, both substances have different effects. CoCl_2_ leads to an early loss of RGCs, which is accompanied by a highly increased apoptosis rate (Kuehn et al., 2017a). Furthermore, CoCl_2_ enhances the expression of several cellular stress markers like, HIF-1α, HSP70, and iNOS (Maliha et al., 2019). Interestingly, cells of the inner nuclear layers, like amacrine and bipolar cells, are damaged in a later point in time by CoCl_2_-induced hypoxia. A further pathway which is strongly induced by CoCl_2_ is apoptosis. Not only the cell cycle arrest gene p21, also caspase 8 and 3, and highly upregulated after the CoCl_2_-treatment (Maliha et al., 2019). A further cell type which is also affected by CoCl_2_, are microglia. CoCl_2_ seems to have toxic effects on microglia, since the attendance of CoCl_2_ induces a prominent loss of them (Kuehn et al., 2017a). Damaging effects through H_2_O_2_ on retinal tissue are similar to those seen after CoCl_2_. The induction of oxidative stress due to H_2_O_2_ led to a strong loss of RGCs, which was associated with higher RGC apoptosis rate. Cells located in the inner nuclear layer, namely bipolar and amacrine cells, were tendentially decreased after oxidative stress. Interestingly, in contrast to the treatment with CoCl_2_, H_2_O_2_ led to a significantly increased microglia cell number as well as induced the activation of these cells. Also, proinflammatory cytokines, such as TNF-α as well as IL-1β, were strongly increased through oxidative stress in retinal organ cultures (Hurst et al., 2017).

The commonly used method for the application of drugs to the eyes are intravitreal injections. Many barriers in the eye itself, like the vitreous body, complicate or even prevent the reaching of the drug to the retina. Here, *ex vivo* bovine eyes can be used to improve or investigate possible enhancements, like nanoparticles, for the drug to reach the retina (Huang et al., 2017; Peynshaert et al., 2017).

Due to the *ex vivo* cultivation, where the retina is separated from the optical nerve and therefore the natural nutrient supply is not taking place anymore, of course, animal experiments cannot be replaced totally by the organ culture. However, the organ culture model in combination with chemical substances, as used in previous studies by our group and others, seems to be a very promising alternative model for analyzing new therapeutic approaches for ophthalmology and might help to reduce the number of animal experiments. Nevertheless, *in vivo* models will still be necessary for the ophthalmic research, not only for investigations of underlying pathomechanisms in several neuroretinal diseases, but also for the proof of principle of newly tested therapeutics. However, the use of *ex vivo* retina organ culture models is increasing, not only used for treatment screening, but also e.g., to study complement involvement in retinal degeneration (Mohlin et al., 2018). In regard to research on new therapeutics, it was shown that autoantibodies, which are downregulated in the vitreous humor of glaucoma patients, seem to have neuroprotective effects on damaged porcine retinae (Bell et al., 2016). In a study by Maliha et al. (2019) it was described that hypoxic processes due to CoCl_2_ can be inhibited by mild hypothermia. Cell-stress level as well as apoptotic mechanisms were strongly diminished after hypothermia treatment, leading to a prominent protection of retinal cells. Even toxic effects of CoCl_2_ on microglia were counteracted by hypothermia. Most interestingly, RGCs, which are affected in glaucoma, were protected by a mild hypothermia due to a significantly decreased apoptosis rate (Maliha et al., 2019).

Taken together, porcine organ culture models seem to be a very promising alternative to conventional animal experiments in the glaucoma research. They are very suitable for the screening of new therapeutic approaches, and therefore can help to reduce the number of animals in the ophthalmic research.

## Conclusion

The results of all these studies undermine that analyzes in different model systems are necessary in order to decode glaucoma pathogenesis in the near future and thus to develop new therapeutic approaches.

Given the high cost of animal testing and their numerous ethical and legal barriers, alternative approaches are becoming increasingly important. Due to the widespread establishment of various organ cultures, *ex vivo* culture systems are presently available also for glaucoma research, since they can be obtained as by-products of the food industry. *Ex vivo* organ cultures, e.g., from porcine retinae, are also quite suitable for therapy testing. Recently, it was shown that a hypothermic treatment protects RGCs from oxidative stress.

Nonetheless, ophthalmic research will continue to require *in vivo* models, not only to investigate the pathomechanisms, but above all to test new therapeutics. It is of enormous importance that the used *in vivo* model reflects the patient’s situation as faithfully as possible. Thus, with regard to glaucoma, it is essential that the various forms of the disease are represented. In the last years, the EAG model was in used to identify mechanisms related to immunological alterations in IOP-independent glaucoma. These animals have a loss of RGCs and optic nerve degeneration plus an enhanced activation of glia cells and complement system proteins. Results point toward the importance especially of the complement system in glaucoma. Immune dysregulation appears to be an important factor for the disease development and progression. Complement and microglia inhibition in the OHT models underlined their potential as future therapeutic approaches. Nevertheless, deeper insights into these mechanisms will lead to better treatment options for glaucoma patients.

## Author Contributions

All authors wrote sections of the manuscript, read and approved the submitted and revised version of the manuscript.

## Conflict of Interest Statement

The authors declare that the research was conducted in the absence of any commercial or financial relationships that could be construed as a potential conflict of interest.

## Abbreviations

CoCl_2_: cobalt-chloride
EAG: experimental autoimmune glaucoma animal
H_2_O_2_: hydrogen peroxide
HSP: heat shock protein
IL: interleukin
IOP: intraocular pressure
L: long
M: middle
MASP1 and 2: the mannose-associated-serine-proteases 1 and 2
MAC: membrane attack complex
MBL: mannose binding lectin
NF κ B: nucleus factor-kappa-light-chain enhancer of activated B cells
NTG: normal-tension glaucoma
OHT: ocular hypertension
ONA: optic nerve antigen homogenate
POAG: primary open-angle glaucoma
RGC: retinal ganglion cells
RPE: retinal pigment epithelium
S: short.
